# Biochemical Characterization of 3-Methyl-4-nitrophenol Degradation in *Burkholderia* sp. Strain SJ98

**DOI:** 10.3389/fmicb.2016.00791

**Published:** 2016-05-25

**Authors:** Jun Min, Yang Lu, Xiaoke Hu, Ning-Yi Zhou

**Affiliations:** ^1^Key Laboratory of Coastal Biology and Bioresource Utilization, Yantai Institute of Coastal Zone Research, Chinese Academy of SciencesYantai, China; ^2^State Key Laboratory of Microbial Metabolism, School of Life Sciences and Biotechnology, Shanghai Jiao Tong UniversityShanghai, China

**Keywords:** 2-chloro-4-nitrophenol, 3-methyl-4-nitrophenol, *Burkholderia* sp. strain SJ98, catabolism, fenitrothion, *para*-nitrophenol

## Abstract

Several strains have been reported to grow on 3-methyl-4-nitrophenol (3M4NP), the primary breakdown product of the excessively used insecticide fenitrothion. However, the microbial degradation of 3M4NP at molecular and biochemical levels remains unknown. Here, methyl-1,4-benzoquinone (MBQ) and methylhydroquinone (MHQ), rather than catechol proposed previously, were identified as the intermediates before ring cleavage during 3M4NP degradation by *Burkholderia* sp. strain SJ98. Real-time quantitative PCR analysis indicated that the *pnpABA1CDEF* cluster involved in *para*-nitrophenol (PNP) and 2-chloro-4-nitrophenol (2C4NP) catabolism was also likely responsible for 3M4NP degradation in this strain. Purified PNP 4-monooxygenase (PnpA) is able to catalyze the monooxygenation of 3M4NP to MBQ and exhibited an apparent *K*_m_ value of 20.3 ± 2.54 μM for 3M4NP, and *pnpA* is absolutely necessary for the catabolism of 3M4NP by gene knock-out and complementation. PnpB, a 1,4-benzoquinone reductase catalyzes the reduction of MBQ to MHQ, and also found to enhance PnpA activity *in vitro* in the conversion of 3M4NP to MBQ. By sequential catalysis assays, PnpCD, PnpE, and PnpF were likely involved in the lower pathway of 3M4NP catabolism. Although NpcCD, NpcE, and NpcF are able to catalyze the sequential conversion of MHQ *in vitro*, these enzymes are unlikely involved in 3M4NP catabolism because their coding genes were not upregulated by 3M4NP induction *in vivo*. These results revealed that the enzymes involved in PNP and 2C4NP catabolism were also responsible for 3M4NP degradation in strain SJ98. This fills a gap in our understanding of the microbial degradation of 3M4NP at molecular and biochemical levels and also provides another example to illustrate the adaptive flexibility in microbial catabolism for structurally similar compounds.

## Introduction

As a representative among the organophosphorus pesticides, fenitrothion (*O*,*O*-dimethyl*O*-*p*-nitro-*m*-tolyl phosphorothioate), is a highly toxic chemical and extensively used to control major insect pests (Hayatsu et al., 2000; Zhang et al., 2006; Itoh et al., 2014), especially in developing countries. Under aerobic environmental conditions, fenitrothion could be degraded rapidly, in days to weeks (Takimoto et al., 1976; Spillner et al., 1979; Mikami et al., 1985), with production of the persistent pollutant 3M4NP, which has serious health effects to humans and animals as an endocrine-disrupting chemical (Furuta et al., 2004; Kanaly et al., 2005; Li et al., 2008, 2009; Verma and Rana, 2009). Naturally occurring bacterial isolates capable of degrading 3M4NP have received considerable attention since they provide the possibility of both environmental and *in situ* detoxification. So far, the microbial degradation of 3M4NP was predominantly reported in *Burkholderia* spp., such as strains SJ98 (originally classified as a *Ralstonia* sp.; Bhushan et al., 2000), SH-1 (Kim et al., 2007), NF100 (Hayatsu et al., 2000), FDS-1 (Zhang et al., 2006), and RKJ800 (Arora and Jain, 2012).

Structurally, 3M4NP is a analog of the priority environmental pollutant PNP and 2C4NP. These nitrophenols compounds are widely used for manufacturing of drugs, dyes, pesticides, herbicides, and fungicides (Arora et al., 2012, 2014). The microbial degradation of PNP (Spain and Gibson, 1991; Jain et al., 1994; Kadiyala and Spain, 1998; Kitagawa et al., 2004; Takeo et al., 2008; Zhang et al., 2009; Liu et al., 2010; Shen et al., 2010; Wei et al., 2010) and 2C4NP (Ghosh et al., 2010; Arora and Jain, 2011, 2012; Pandey et al., 2011; Min et al., 2014, 2016) has been extensively investigated. For PNP degradation, the hydroquinone pathway was initiated by a single-component PNP monooxygenase (Zhang et al., 2009; Shen et al., 2010; Wei et al., 2010) and the hydroxyquinol pathway was initiated by a two-component PNP monooxygenase (Kadiyala and Spain, 1998; Kitagawa et al., 2004; Takeo et al., 2008; Liu et al., 2010). Recently, the enzymes encoded by *pnpABCDEF* were proved to be involved in the chlorohydroquinone pathway of 2C4NP catabolism in Gram-negative *Burkholderia* sp. SJ98 (Min et al., 2014) and the enzymes encoded by *pnpA1A2BC* were proved to be responsible for the hydroxyquinol pathway of 2C4NP catabolism in Gram-positive *Rhodococcus imtechensis* RKJ300 (Min et al., 2016).

In contrast to PNP and 2C4NP, the knowledge of the microbial degradation of 3M4NP is limited, with no genetic or biochemical investigation being reported. So far, two different pathways based on different intermediates present during 3M4NP degradation have been proposed in above five 3M4NP utilizers. Strains SJ98 (Bhushan et al., 2000) and SH-1 (Kim et al., 2007) were reported to degrade 3M4NP with catechol as an intermediate, revealing that the methyl group of 3M4NP was removed before ring cleavage. However, strains RKJ800 (Arora and Jain, 2012), NF100 (Hayatsu et al., 2000) and FDS-1 (Zhang et al., 2006) were reported to degrade 3M4NP with MHQ as the ring cleavage substrate, indicating that removal of the methyl group occurs after ring cleavage in these three strains. However, none of these two alternative pathways has been characterized at the genetic and enzymatic levels, either for the initial degardation or the ring cleavge reaction.

*Burkholderia* sp. strain SJ98 was previously reported to utilize 3M4NP with catechol as the intermediates (Bhushan et al., 2000), but with no genetic and enzymatic inverstigation. The goal of this study was to inverstigate the microbial degradation of 3M4NP by strain SJ98 at molecular and biochemical levels. To our surprise, MBQ and MHQ, rather than catechol were identified as the intermediates before ring cleavage during 3M4NP degradation by this strain. On the other hand, the enzymes encoded by the *pnpABA1CDEF* cluster were proved to be also responsible for 3M4NP degradation by this strain, in addition to PNP and 2C4NP degradation (Min et al., 2014). This study fills a gap in our understanding of the microbial degradation mechanism of 3M4NP at the biochemical and genetic levels and also provides another example to illustrate the adaptive flexibility in microbial catabolism for structurally similar compounds. Considering that strain SJ98 is capable of degrading PNP as well as its chloro- and methyl-substituted derivatives, it is reasonable to conclude that it is of potential in bioremediation of these toxicants-contaminated sites.

## Materials and Methods

### Bacterial Strains, Plasmids, Primers, Media, and Culture Conditions

The bacterial strains and plasmids used in this study are described in **Table 1**, and the primers used are listed in **Table 2**. *Escherichia coli* strains were grown in lysogeny broth (LB) at 37°C. *Burkholderia* strains were grown at 30°C in minimal medium (MM; Xiao et al., 2006) supplemented with 0.5 mM 3M4NP (2 mM glucose was added to enhance the biomass when cultures were prepared for biotransformation assays). When necessary, kanamycin at 50 mg/ml was added to the medium.

**Table 1:** Bacterial strains and plasmids used in this study.

Strain or plasmid	Relevant genotype or characteristic(s)	Reference
***Burkholderia* sp.**		
SJ98	3M4NP, PNP, and 2C4NP utilizer, wild type	Min et al., 2014
SJ98Δ*pnpA*	SJ98 mutant with *pnpA* gene deleted	Min et al., 2014
SJ98Δ*pnpA*1	SJ98 mutant with *pnpA1* gene deleted	Min et al., 2014
SJ98Δ*pnpA* [pRK415-*pnpA*]	*pnpA* gene was complemented by pRK415-*pnpA* in SJ98Δ*pnpA*	Min et al., 2014
***Escherichia coli* strains**		
DH5α	*supE44 lacU169 (*φ80*lacZ*ΔM15*) recA1 endA1 hsdR17 thi-1 gyrA96 relA1*	Novagen
Rosetta(DE3)pLysS	F^-^*ompThsdS*(r_B_^-^m_B_^+^) *gal dcm*, *lacY1*(DE3) pLysSRARE (Cm^r^)	Novagen
**Plasmids**		
pET-28a	Expression vector, Kan^R^	Novagen
pET-*pnpA*	*Nde*I-*Xho*I fragment containing *pnpA* inserted into pET-28a	Min et al., 2014
pET-*pnpB*	*Nde*I-*Xho*I fragment containing *pnpB* inserted into pET-28a	Min et al., 2014
pET-*pnpCD*	*Nde*I-*Xho*I fragment containing *pnpCD* inserted into pET-28a	Min et al., 2014
pET-*pnpE*	*EcoR*I-*Xho*I fragment containing *pnpE* inserted into pET-28a	Min et al., 2014
pET-*pnpF*	*Nde*I-*Xho*I fragment containing *pnpF* inserted into pET-28a	Min et al., 2014
pET-*npcCD*	*Nde*I-*Xho*I fragment containing *npcCD* inserted into pET-28a	This study
pET-*npcE*	*Nde*I-*Xho*I fragment containing *npcE* inserted into pET-28a	This study
pET-*npcF*	*Nde*I-*Xho*I fragment containing *npcF* inserted into pET-28a	This study

**Table 2:** Primers used in this study.

Primers	Sequence (5′–3′)*	Purpose and Reference
*npcCD*-F	AGGCACCATATGGAGACAGACATGCAACAG	To amplify *npcCD* gene for expression
*npcCD*-R	GGACTCGAGGAACGCGACCGGATACG	
*npcE*-F	AGGCACCATATGCAAACGCAACTCTTCATC	To amplify *npcE* gene for expression
*npcE*-R	GGACTCGAGTCAACGTGGATAGTAAGGCGG	
*npcF*-F	AGGCACCATATGCAATCGTTCGTTTATCAGGGC	To amplify *npcF* gene for expression
*npcF*-R	GGACTCGAGTCATGGTCGTCGTCCTTCGTAG	
RTCD-F	CGAAGGCTCGGTGAAACTC	To amplify 456 bp of the *npcC*-*npcD* spanning region
RTCD-R	CCAGCCGTAGAAGAAACC	
RTDE-F	ATCCGCCACAAGGGTTATTC	To amplify 429 bp of the *npcD*-*npcE* spanning region
RTDE-R	GTCGGCGAGTTTCAGGAGC	
RTEF-F	GATACGGACGCGAGATGG	To amplify 179 bp of the *npcE*-*npcF* spanning region
RTEF-R	CGATGCTTCCCGCACCG	
RTFG-F	ATTGAACGCCGACGATGC	To amplify 397 bp of the *npcF*-*npcG* spanning region
RTFG-R	GGATGCCGTCGCACTTCTTG	
RTq16S-F	CGTGTAGCAGTGAAATGCGTAGAG	Min et al., 2014
RTq16S-R	GACATCGTTTAGGGCGTGGAC	
RTq-*pnpA*-F	CGTCGCAACGAATGTCTTCTATG	Min et al., 2014
RTq-*pnpA*-R	CATACGACGACGCACTTCCTC	
RTq-*npcC*-F	CGAAGGCTCGGTGAAACTC	To amplify a 134 bp fragment of *npcC* for RT-qPCR
RTq-*npcC*-R	GCCCATTTCTCGACCGATTC	

*Specified restriction sites are underlined.

### Biotransformation and Intermediates Identification

Biotransformation of 3M4NP by strain SJ98 was performed as previously described for PNP and 2C4NP (Min et al., 2014). In order to accumulate the intermediates before ring cleavage, 1 mM 2, 2′-dipyridyl, a known inhibitor for ferrous-dependent aromatic ring dioxygenases (Chapman and Hopper, 1968; Kadiyala and Spain, 1998; Ferreira et al., 2008), was added into the biotransformation mixtures. Quantitative analyses of 3M4NP and its catabolic intermediates were performed by high performance liquid chromatography (HPLC) analysis. One unit of activity was defined as the amount of cell (milligram of cell dry weight) required to transform 1 μmol of 3M4NP per min at 30°C. For gas chromatography-mass spectrometry (GC-MS) analysis of the intermediates, the supernatant was extracted with ether after acidification, and the extract was then dried over sodium sulfate.

### Analytical Methods

High performance liquid chromatography analysis was performed with an Agilent 1200 system (Agilent Technologies, Palo Alto, CA, USA) equipped with a diode array detector and an Agilent ZORBAX Eclipse XDB-C18 column (250 mm × 4.6 mm, 5 μm particle size). The mobile phase consisted of solvents A (0.1% acetic acid in water) and B (methanol) with a gradient program started with 10% of B, followed by increasing to 90% B from 0 to 12 min, hold at 90% B from 12 to 15 min, then back to 10% B in 0.1 min and equilibrate for 2.9 min. The flow rate was 1.0 ml/min, and the injection volume was 10 μl. MBQ, MHQ, and 3M4NP were quantified at 254, 290, and 320 nm, respectively. Under these conditions, authentic MBQ, MHQ, and 3M4NP had retention times of 11.7, 8.9, and 13.5 min, respectively.

Gas chromatography-mass spectrometry was performed using a Thermo Fisher Trace GC Ultra gas chromatograph equipped with an HP-5MS capillary column (30 m × 0.25 mm × 0.25 μm) and coupled to a Thermo Fisher ITQ 900 ion trap mass spectrometer. The conditions used for GC-MS analysis were the same as those described previously (Zhang et al., 2009). Under these conditions, authentic MBQ and MHQ had GC retention times of 10.06 and 16.05 min, respectively. The intermediates were identified based on comparisons of the mass spectra with those of authentic compounds and those available in an NIST98 MS data library, in addition to comparing the GC retention times of the intermediates with those of the authentic compounds.

High performance liquid chromatography-MS analysis was carried out on a Dionex UltiMate 3000 RS HPLC system coupled to an LCQ Fleet^TM^ ion trap mass spectrometer (Thermo Fisher Scientific, Waltham, MA, USA). Chromatographic separation was carried out on a Hypersil GOLD column (150 × 4.6 mm, 3 μm particle size) using gradient elution. The mobile phase consisted of solvents A (water) and B (acetonitrile) as follows: 10% B from 0 to 5 min; increasing to 60% B from 5 to 20 min, hold at 60% B from 20 to 25 min, then back to10% B in 0.1 min and equilibrate for 1.9 min. The flow rate was 0.8 ml/min. The mass spectrometer was operated in the negative ion mode and full scan (40–300 m/z). The sample solutions were nebulized and evaporated using nitrogen as the sheath and auxiliary gas at the flow rate of 35 and 10 arb (1 arb = 0.3 l/min), respectively. The ion spray voltage was set at 5 kV, and the transfer capillary temperature was maintained at 300°C.

### Real-Time Quantitative PCR

The total RNA was isolated with an Easy Pure RNA Kit (TransGen Biotech, Beijing, China) and reverse transcribed into cDNA using a TransScript One-Step gDNA Removal and cDNA Synthesis SuperMix Kit (TransGen). Reverse transcription PCR was carried out with the primers described in **Table 2**. Transcriptional analysis was carried out in order to investigate whether the *pnpABA1CDEF* (Min et al., 2014) and *pnpE2E1FD* clusters (Vikram et al., 2012) involved in PNP catabolism in strain SJ98 were highly transcribed in response to 3M4NP. Real-time quantitative PCR (RT-qPCR) was performed on a 7500 Fast Real-Time PCR System (Applied Biosystems) using TransStart Tip Green qPCR SuperMix (TransGen) with primers in **Table 2**. All samples were run in triplicate in three independent experiments. Relative expression levels were estimated using the 2^-ΔΔC_T_^ method (Livak and Schmittgen, 2001), and the 16S rRNA gene was used as a reference for normalization.

### Protein Expression and Purification

N-terminal His-tagged Pnp proteins (H_6_-PnpA, H_6_-PnpB, H_6_-PnpCD, H_6_-PnpE, and PnpF) were expressed by *E. coli* Rosetta(DE3)pLysS carrying the corresponding expression plasmids constructed previously (Min et al., 2014). In order to express the N-terminal His-tagged Npc proteins, *npcCD*, *npcE*, and *npcF* were amplified using the primers listed in **Table 2**, digested with *Nde*I and *Xho*I, and inserted into pET-28a to obtain expression plasmids. The plasmids were then transformed into *E. coli* Rosetta(DE3)pLysS for protein expression and purification as described (Liu and Zhou, 2012).

### Enzyme Assays

The activity assays of PNP 4-monooxygenase (PnpA) against 3M4NP and 1,4-benzoquinone reductase (PnpB) against MBQ were performed as previously described for these two enzymes against 2C4NP and 2-chloro-1,4-benzoquinone, respectively (Min et al., 2014). Identification of the products from H_6_-PnpA or H_6_-PnpB-catalyzed reactions was carried out by HPLC and GC-MS analysis as described previously (Zhang et al., 2009). Enzyme activity assays of hydroquinone dioxygenase (PnpCD and NpcCD), 4-hydroxymuconic semialdehyde dehydrogenase (PnpE and NpcE) and maleylacetate reductase (PnpF and NpcF) involved in the sequential transformation of MHQ were performed as previously described for sequential transformation of hydroquinone and chlorohydroquinone (Min et al., 2014).

In the case of the kinetics assays of H_6_-PnpA against 3M4NP, 7 concentrations of 3M4NP raging from 5 to 100 μM were used while the concentration of NADPH was fixed at 400 μM. Data from three independent sets of experiments were fitted with the Michaelis–Menten equation by OriginPro 8 software. The protein concentration was determined according to the Bradford method (Bradford, 1976) with bovine serum albumin as a standard. One unit of enzyme activity was defined as the amount of protein required to catalyze the conversion of 1 μmol of 3M4NP per min at 30°C. The nitrite concentration was determined as previously described (Lessner et al., 2002).

## Results

### Identification of MBQ and MHQ as Metabolites

**FIGURE 1:**
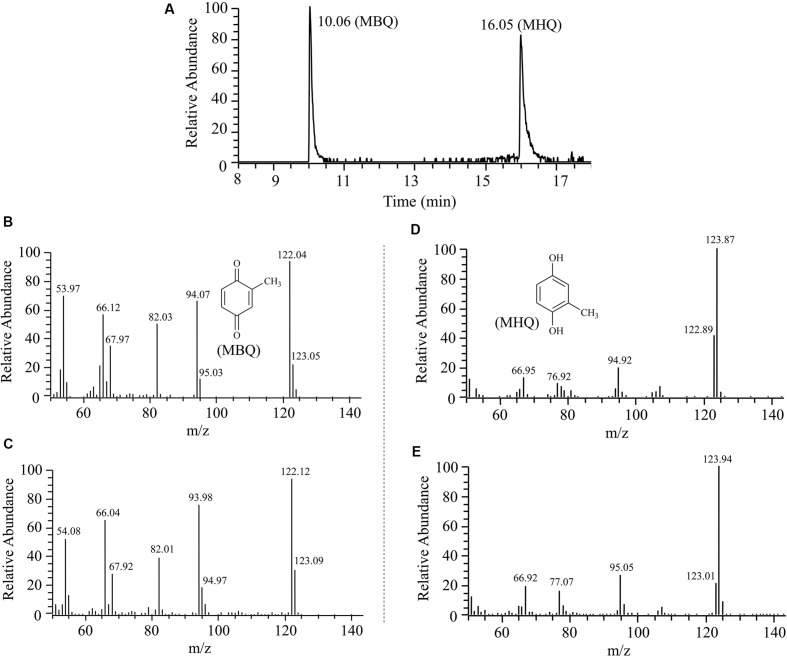
**Gas chromatography-mass spectrometry (GC-MS) analysis of the intermediates captured during 3M4NP degradation by *Burkholderia* sp. strain SJ98. (A)** The gas chromatogram is the extracted ion current chromatogram at m/z 122.00 ± 0.50 and 124.00 ± 0.50 from the total ion current chromatogram. **(B)** Mass spectrum of authentic methyl-1,4-benzoquinone (MBQ). **(C)** Mass spectrum of the GC peak at 10.06 min, identical to that of authentic MBQ. **(D)** Mass spectrum of authentic methylhydroquinone (MHQ). **(E)** Mass spectrum of the GC peak at 16.05 min, identical to that of authentic MHQ.

**FIGURE 2:**
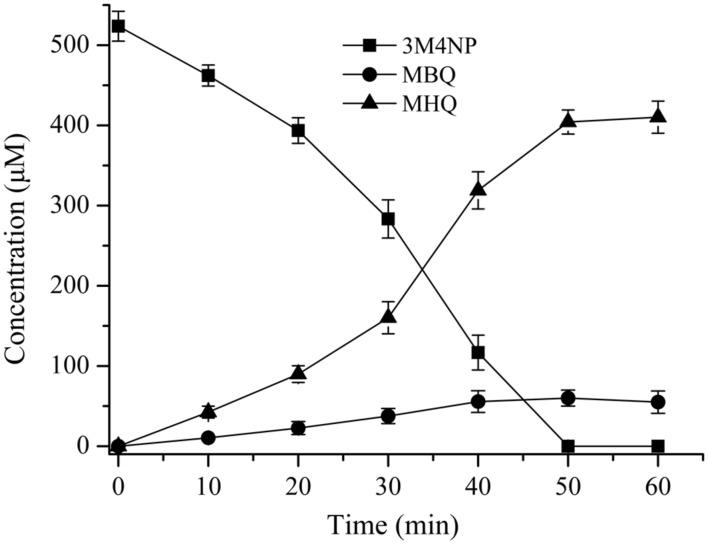
**Time course of 3M4NP degradation by *Burkholderia* sp. strain SJ98 in whole cell biotransformation.** In order to accumulate the intermediates before ring cleavage, 1 mM 2, 2′-dipyridyl was added to inhibit the ring cleavage enzyme. The disappearance of 3M4NP and the appearance of the products (MBQ and MHQ) were quantified by HPLC. The experiments were performed in triplicate; the results were the average of three independent experiments, and error bars indicate standard deviations.

**FIGURE 3:**
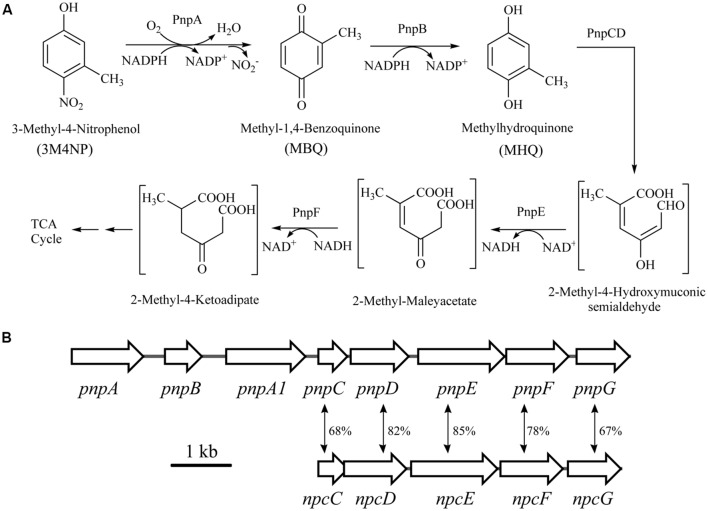
**(A)** Proposed pathways for 3M4NP catabolism in *Burkholderia* sp. strain SJ98, together with the catabolic reactions catalyzed by *pnp* gene products. **(B)** Organization of the *pnp* and *npc* gene clusters of strain SJ98. The large black arrows indicate the sizes and directions of transcription of each gene or ORF. The double-headed arrows demonstrated the alignment of the catabolic genes with significant similarities. Percentage identities at the amino acid level are indicated adjacent.

The intermediates of 3M4NP catabolism were identified by HPLC analysis after the confirmation of strain SJ98 as a 3M4NP utilizer. Initially, no metabolite was captured during 3M4NP degradation. Therefore, 2, 2′-dipyridyl was added to the biotransformation system in order to accumulate the intermediates before ring cleavage. In this way, two intermediates with HPLC retention times of 11.7 and 8.9 min, respectively, were captured during 3M4NP degradation. The retention times of these two metabolites matched precisely to that of the standard MBQ and MHQ. Moreover, the identification of MBQ and MHQ was also confirmed by GC-MS analysis by comparison with mass spectra of the authentic compounds (**Figure 1**). In a time course assay of biotransformation, 3M4NP consumption (523 μM) was approximately equivalent to the total accumulation of both MBQ (55 μM) and MHQ (410 μM) (**Figure 2**), indicating a nearly stoichiometric formation of MBQ and MHQ from 3M4NP. Hence, the identification of metabolites clearly indicated that strain SJ98 degraded 3M4NP with MBQ and MHQ as the intermediates before ring cleavage (**Figure 3A**).

### Biotransformation of 3M4NP by Strain SJ98

The uninduced cells of strain SJ98 exhibit negligible activity for 3M4NP. However, the 3M4NP-induced cells exhibited a specific activity of 3.27 ± 0.46 U mg^-1^ for 3M4NP, indicating that the enzymes involved in 3M4NP degradation in strain SJ98 are inducible. Interestingly, PNP-induced cells degrade 3M4NP (3.56 ± 0.64 U mg^-1^) while 3M4NP-induced strain SJ98 also have the ability to degrade PNP (5.56 ± 0.84 U mg^-1^). This finding indicated that the enzymes involved in PNP catabolism were likely also responsible for 3M4NP degradation by strain SJ98.

### Transcriptional Analysis of the *pnpABA1CDEF* and *npcCDEF* Clusters

**FIGURE 4:**
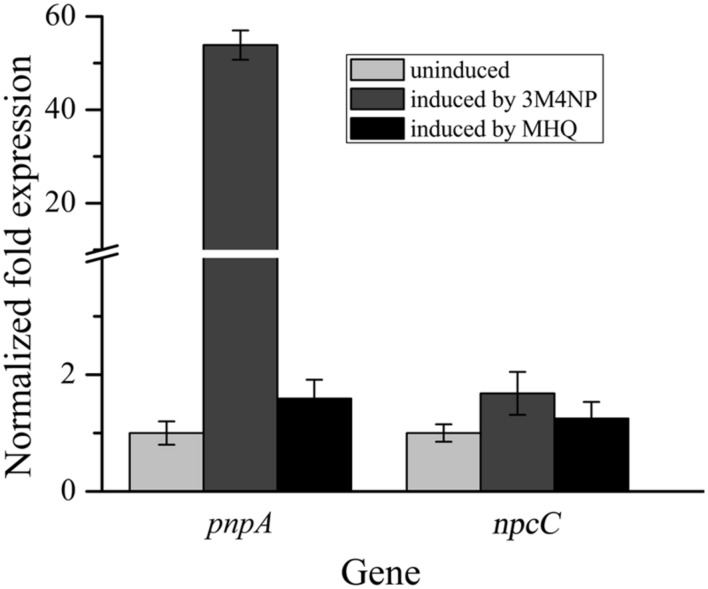
**Transcriptional analyses of *pnpA* and *npcC* in *Burkholderia* sp. strain SJ98 under induced and uninduced conditions by RT-qPCR.** The data are derived from three independent measurements, and error bars indicate standard deviations.

In order to distinguish the *pnpABA1CDEF* and *pnpE2E1FD* clusters named originally in strain SJ98, the *pnpE2E1FD* cluster was tentatively renamed as *npcCDEF* in this study (**Figure 3B**). Considering that the genes of both clusters were in a single transcriptional operon, respectively, the transcriptional analyses of *pnpA* and *npcC* (each representing the operons they belong to) were carried out under various induction conditions by real-time quantitative PCR. The transcription level of *pnpA* under 3M4NP-induced condition was enhanced dramatically compared to that from the non-induced sample, with 54-fold increase (**Figure 4**), suggesting the involvement of Pnp proteins in 3M4NP catabolism. In contrast to *pnpABA1CDEF* cluster, the transcription level of the *npcCDEF* cluster was not increased by 3M4NP induction (**Figure 4**). On the other hand, no transcription increase of *pnpA* was observed by MHQ induction, a metabolite in 3M4NP catabolism.

### PnpA-Catalyzed Monooxygenation of 3M4NP to MBQ

**FIGURE 5:**
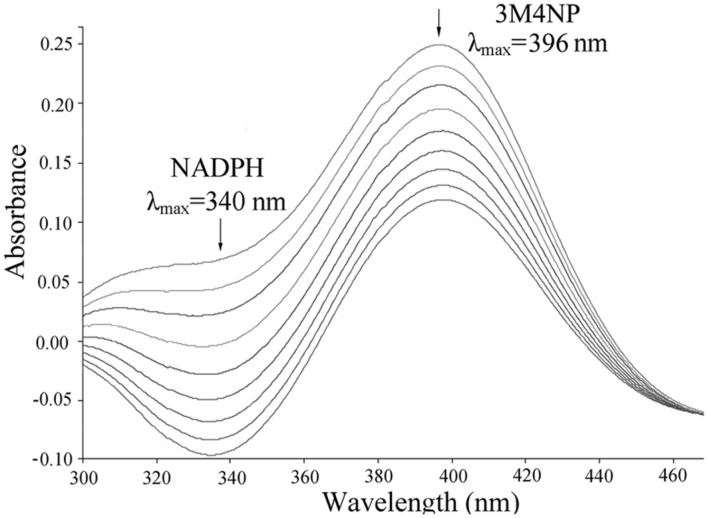
**Spectral changes during the transformation of 3M4NP by H_6_-PnpA.** Sample and reference cuvettes contained 0.1 mM NADPH, 0.03 mM FAD, 50 mM phosphate buffer (pH 7.4), and 20 μg H_6_-PnpA in a 0.5-ml mixture. The reaction was initiated by addition of 30 μM 3M4NP, and the spectra were recorded every minute after the addition of 3M4NP. The arrows indicate the directions of spectral changes.

*Escherichia coli* cells carrying pET-*pnpA* was found to be able to degrade 3M4NP by HPLC analysis, along with the stoichiometric release of nitrite ion. In contrast, neither 3M4NP consumption nor nitrite ion release was detected in the negative control when the cells harboring only pET-28a vector. The PnpA activity measured spectrophotometrically showed that the purified H_6_-PnpA catalyzed rapid degradation of 3M4NP (λ_max_, 396 nm) with a specific activity of 3.36 U mg^-1^, together with consumption of NADPH (λ_max_, 340 nm; **Figure 5**). By HPLC and GC-MS analyses, both MBQ and MHQ were detected as the products from 3M4NP monooxygenation catalyzed by the purified H_6_-PnpA. Kinetics assays revealed that the *K*_m_ value of H_6_-PnpA for 3M4NP was 20.3 ± 2.54 mM.

### PnpB-Catalyzed Reduction of MBQ to MHQ

**FIGURE 6:**
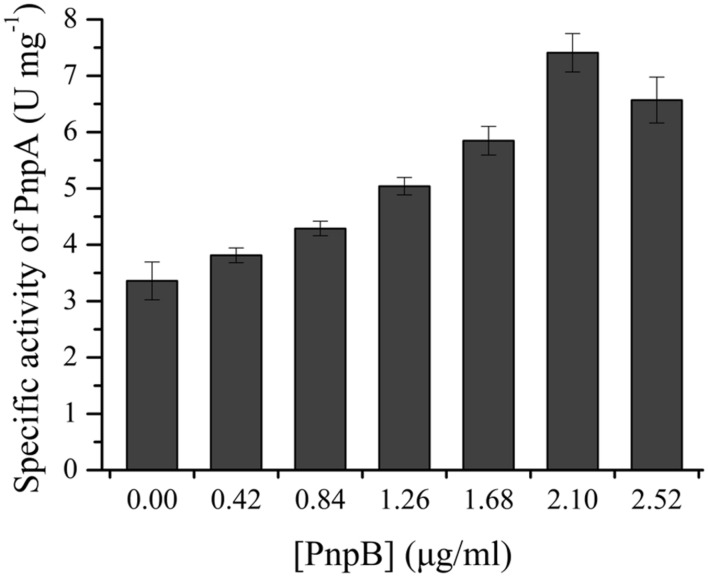
**H_6_-PnpB enhanced the 3M4NP degradation by H_6_-PnpA.** The reaction mixtures contained 0.1 mM NADPH, 0.03 mM FAD, 50 mM phosphate buffer (pH 7.4), 20 μg H_6_-PnpA and different amount of H_6_-PnpB (0–2.52 μg) in a 0.5 ml mixture. The reaction was initiated by addition of 30 μM 3M4NP.

The activity assay of PnpB against MBQ was carried out in order to confirm the presence of MBQ in the 3M4NP degradation. By HPLC analysis, the product of MBQ catalyzed by the purified H_6_-PnpB was identified as MHQ. Moreover, when H_6_-PnpB was added to a reaction mixture containing H_6_-PnpA, a clear increase in total 3M4NP monooxygenase activity was observed (**Figure 6**). A likely explanation for the enhanced activity was that PnpB reduced the formed MBQ to MHQ, probably preventing product inhibition of PnpA activity. Particularly, the increased 3M4NP monooxygenase activity with H_6_-PnpB further proves the formation of MBQ during 3M4NP catabolism *in vivo*, apart from the HPLC and GC-MS identification of MBQ as a metabolite.

### PnpCD-, PnpE-, and PnpF-Catalyzed Sequential Reactions from MHQ in the Lower Pathway

**FIGURE 7:**
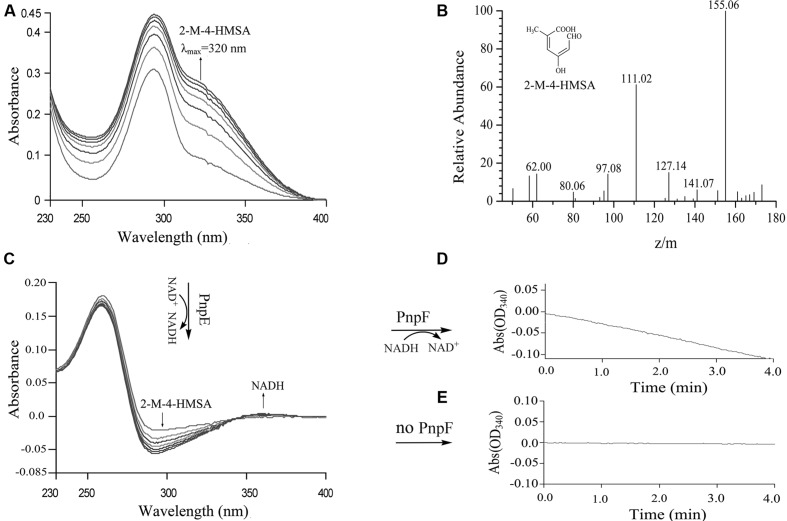
**Enzyme activity assays of PnpCD, PnpE, and PnpF by sequential catalytic reactions with MHQ as the starting substrate. (A)** Spectral changes during the transformation of MHQ by H_6_-PnpCD. Sample and reference cuvettes contained 0.04 mM Fe^2+^, 5 μg H_6_-PnpCD and 20 mM phosphate buffer (pH 7.4) in a 1 ml mixture. The reaction was initiated by the addition of 0.1 mM MHQ. **(B)** Mass spectrum of the product of MHQ catalyzed by purified PnpCD. The reaction mixture of MHQ catalyzed by H_6_-PnpCD was extracted with ethyl acetate, and the product was then identified by HPLC-MS analysis. **(C)** Enzyme activity assay of PnpE. Following the complete oxidation of MHQ by H_6_-PnpCD, the sample cuvette from the reaction in A was added by 50 μM NAD^+^, and then divided into two cuvettes (sample and reference). The spectra were recorded every minute after the addition of 20 μg H_6_-PnpE. **(D,E)** Enzyme activity assay of PnpF. Following the complete conversion of 2-methyl-4-hydroxymuconic semialdehyde (2-M-4-HMSA) to 2-methylmaleylacetate, the sample cuvette from the reaction in C was added by 50 μM NADH, and then divided into two cuvettes (sample and reference). H_6_-PnpF (20 μg; **D)** or buffer without H_6_-PnpF **(E)** was added to initiate the assay.

When purified His_6_-PnpCD was incubated with MHQ, a spectral change from 290 to 320 nm occurred to form a new compound with a λ_max_ of 320 nm (**Figure 7A**). In contrast, no spectral change was observed when His_6_-PnpCD was omitted from the mixture. By HPLC-MS analysis, the ring cleavage product of MHQ was found to have a retention time of 6.63 min and proposed as 2-methyl-4-hydroxymuconic semialdehyde, with the deprotonated ion peak at m/z 155.06 and its fragments at m/z 111.02 (loss of -COOH), at m/z 127.14 (loss of -CHO), and at m/z 141.07 (loss of –CH_3_) (**Figure 7B**).

The activity assays of His_6_-PnpE and His_6_-PnpF were carried out by sequential catalyzes in order to investigate whether these two enzymes were also involved in 3M4NP degradation. The substrate of PnpE was 2-methyl-4-hydroxymuconic semialdehyde formed from the above PnpCD-catalyzed dioxygenation of MHQ. The absorbance of 2-methyl-4-hydroxymuconic semialdehyde (λ_max_, 320 nm) became progressively lower after addition of His_6_-PnpE, together with the production of NADH (**Figure 7C**), presumably forming 2-methylmaleylacetate. Subsequently, the reaction mixture of PnpE-catalyzed reaction was used to assay the activity of PnpF. As shown in **Figure 7D**, NADH was consumed gradually upon the addition of purified PnpF to the assay mixture, indicating that PnpF has 2-methylmaleylacetate reduction activity, presumably producing 2-methy-4-ketoadipate (**Figure 3A**). However, no oxidation of NADH was observed when H_6_-PnpF was omitted from the reaction mixture (**Figure 7E**). Although the transcription level of *npc* cluster has no apparent increase after 3M4NP induction, the purified His_6_-NpcCD, His_6_-NpcE, and His_6_-NpcF were also found to have the ability to catalyze the sequential conversion of MHQ, and the spectra changes are similar to those shown in **Figure 7**.

### The Crucial Role of *pnpA* in 3M4NP Catabolism

**FIGURE 8:**
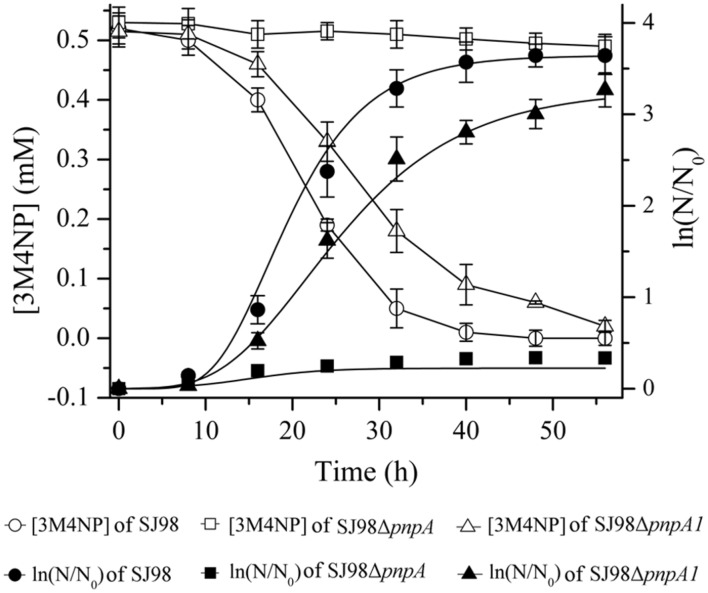
**Time course of 3M4NP degradation and cell growth in cultures of *Burkholderia* sp. strains SJ98, SJ98Δ*pnpA* and SJ98Δ*pnpA1*.** N, numberof cells; N_0_, initial number of cells. All of the experiments were performed in triplicate; the results were the average of three independent experiments, and error bars indicate standard deviations. The data of *pnpA*-complemented mutant SJ98Δ*pnpA*[pRK415-*pnpA*] were not shown but similar to those of *Burkholderia* sp. strain SJ98.

Strains SJ98Δ*pnpA* (with *pnpA* deleted) and SJ98Δ*pnpA1* (with *pnpA1* deleted) described in **Table 1** were, respectively, used to investigate the physiological roles of *pnpA* and *pnpA1* in 3M4NP degradation *in vivo*. Strain SJ98Δ*pnpA* was no longer to grow with 3M4NP as sole carbon and energy source, and *pnpA*-complemented mutant SJ98Δ*pnpA*[pRK415-*pnpA*] regained its ability to utilize 3M4NP (**Figure 8**). This indicated that *pnpA* is absolutely essential for strain SJ98 to utilize 3M4NP. In contrast, although strain SJ98Δ*pnpA1* still has the ability to grow on 3M4NP, its maximum specific growth rate (μ_m_, 0.154 h^-1^) is approximately 28% lower than that of wild-type strain SJ98 (μ_m_, 0.214 h^-1^), in addition to a slower rate of 3M4NP removal (9.3 μM h^-1^ for SJ98Δ*pnpA1*, compared with 13.0 μM h^-1^ for the wild type strain; **Figure 8**). This indicated that *pnpA1* makes a partial contribution in 3M4NP catabolism in strain SJ98, but it is not necessary in 3M4NP catabolism and cannot maintain the cell growth alone.

## Discussion

Considering that fenitrothion could be degraded rapidly to 3M4NP in the environment (Takimoto et al., 1976; Spillner et al., 1979; Mikami et al., 1985), the contamination in the fenitrothion-treated environmentis is more likely due to the presence of 3M4NP rather than fenitrothion itself. In recent years, the microbial degradation of 3M4NP has been attracting considerable attentions (Bhushan et al., 2000; Hayatsu et al., 2000; Zhang et al., 2006; Kim et al., 2007; Arora and Jain, 2012). *Burkholderia* sp. strain SJ98, previously identified as *Ralstonia* sp. SJ98 was proposed to degrade 3M4NP with catechol as a metabolite (Bhushan et al., 2000), with no genetic and enzymatic evidences. However, catechol was not detected as the metabolites of 3M4NP degradation by either HPLC or GC-MS analyses. Furthermore, quantitative analysis of the intermediates have shown that strain SJ98 degrade 3M4NP with MBQ and MHQ as the metabolites before ring cleavage, the same as that reported in *Burkholderia* sp. strains NF100 (Hayatsu et al., 2000), FDS-1 (Zhang et al., 2006) and RKJ800 (Arora and Jain, 2012). Subsequent biochemical and genetic analyses fill a gap in our knowledge of the 3M4NP degradation mechanism at the molecular and biochemical levels in a bacterial strain.

Recently, the enzymes encoded by the *pnpABA1CDEF* cluster were reported to be responsible for the catabolism of PNP and 2C4NP in strain SJ98 (Min et al., 2014). In this study, the high transcription of the *pnpABA1CDEF* cluster in the 3M4NP-induced cell indicated that the enzymes encoded by the cluster were also likely involved in 3M4NP catabolism in this strain. Enzymatic assay has shown that PnpA has the ability to catalyze the monooxygenation of 3M4NP, and its encoding gene is absolutely necessary for strain SJ98 to grow on 3M4NP. Although both MBQ and MHQ were detected during 3M4NP degradation by purified PnpA, the direct product should be MBQ because it is generally accepted that monooxygenases produce a quinone after removal of an electron-withdrawn nitro group from a phenolic compound (Haigler et al., 1996; Perry and Zylstra, 2007; Liu et al., 2010; Min et al., 2014). The detection of MHQ is probably due to the non-enzymatic reaction of MBQ in the presence of NADPH, and the same explanations were also proposed previously for PNP (Perry and Zylstra, 2007; Zhang et al., 2009), 2C4NP (Min et al., 2014, 2016) and 2,4,6-trichlorophenol (Xun and Webster, 2004) monooxygenation. Moreover, the enhancement of 3M4NP monooxygenase activity of PnpA by PnpB also confirms the involvement of MBQ during 3M4NP degradation by strain SJ98. The enhanced activity of PnpA is likely due to the reduction of MBQ to MHQ by PnpB, possibly preventing product inhibition of PnpA. Previously, the analogous phenomena were also observed during monooxygenation of PNP (Zhang et al., 2009), 2C4NP (Min et al., 2014) and 2,4,6-trichlorophenol (Belchik and Xun, 2008) in the presence of corresponding quinone reductases. On the other hand, although PnpA catalyzes the monooxygenation of 3M4NP, in addition to PNP and 2C4NP (Min et al., 2014), its *K*_m_ value for 2C4NP (6.2 ± 0.76 mM reported previously) is evidently lower than those for 3M4NP (20.3 ± 2.54 mM) and PNP (25.4 ± 3.63 mM reported previously), implying that 2C4NP is the probable physiological substrate for PnpA in strain SJ98.

Although *Burkholderia* sp. strains NF100 (Hayatsu et al., 2000), FDS-1 (Zhang et al., 2006), and RKJ800 (Arora and Jain, 2012), were also proposed to degrade 3M4NP with MHQ as the ring cleavage substrate, MBQ was not actually detected and the fate of MHQ was not revealed in these cases. In current study, PnpCD was found to be able to catalyze the dioxygenation of MHO, presumably forming 2-methyl-4-hydroxymuconic semialdehyde (**Figure 3A**). This is similar to previous reports in which the hydroquinone dioxygenase (HapCD) from *Pseudomonas fluorescens* ACB was also reported to be able to catalyze the dioxygenation of MHQ to methyl substituted 4-hydroxymuconic semialdehyde (Moonen et al., 2008). PnpE and PnpF were found to be able to catalyze the sequential conversion of 2-methyl-4-hydroxymuconic semialdehyde, presumably forming 2-methylmaleylacetate and 2-methy-4-ketoadipate, respectively. Previously, a maleylacetate reductase from *Pseudomonas* sp. strain B13 was also found to have the ability to catalyze the reduction of 2-methylmaleylacetate (Kaschabek and Reineke, 1993, 1995). Although NpcCD, NpcE, and NpcF also have the ability to catalyze the sequential transformation of MHQ *in vitro*, their coding genes were not upregulated by 3M4NP induction. Therefore, it is reasonable to conclude that NpcCD, NpcE, and NpcF are unlikely involved in the catabolism of 3M4NP in this strain.

It is generally thought that all the enzymes involved in the complete catabolic pathway of a compound are able to catalyze the initial transformation of this compound, as well as the subsequent reactions of all formed intermediates, to simple organic acids channeled into TCA cycle. Interestingly, the catabolism of PNP, 2C4NP, and 3M4NP by strain SJ98 of the current study are catalyzed by the same complete set of enzymes encode by the single *pnpABA1CDEF* cluster. This indicated that all of the Pnp enzymes exhibited extended substrate specificity for every single metabolites (chlorinated or methylated), resulting in its growth on all these three nitrophenols. In contrast, the Gram-positive PNP and 2C4NP utilizer *R. imtechensis* RKJ300, with a two-component PNP monooxygenase to initiate 2C4NP and PNP catabolism (Min et al., 2016), was found to be unable to grow on 3M4NP in this study. This could be due to several reasons, such as: (i) PnpA1A2 or other enzymes are inactive toward 3M4NP or methylated metabolites in the pathway; or (ii) inability of 3M4NP to induce the expression of the enzymes. During the investigation of *ortho*-nitrophenol (ONP) degradation by *P. putida* B2, the ONP monooxygenase from this strain has the ability to catalyze the monooxygenation of ONP, 4-chloro-2-nitrophenol and 4-methyl-2-nitrophenol to the ring-cleavage substrates catechol, 4-chloro-catechol and 4-methyl-catechol, respectively, but the ring-cleavage catechol dioxygenase in this strain virtually has no activity toward 4-chloro-catechol (Zeyer et al., 1986). However, the ring-cleavage enzyme PnpCD from strain SJ98 is able to efficiently catalyze the dioxygenation of hydroquinone, chlorohydroquinone (Min et al., 2014) and MHQ, the products of PNP, 2C4NP, and 3M4NP, respectively. Moreover, the enzymes involved in ONP catabolism in strain B2 only induced by unsubstituted ONP but not chloro- and methyl-substituted ONPs, whereas the enzymes involved in PNP catabolism in strain SJ98 is induced by PNP, 3M4NP (methyl-PNP) and 2C4NP (chloro-PNP).

Despite that the detailed genetic determinant of 3M4NP catabolism in *Burkholderia* sp. strain NF100 was unknown, previous plasmid curing experiments indicated that the genes involved in the conversion of 3M4NP to MHQ were located on the chromosome, whereas the genes responsible for MHQ degradation were on the plasmid pNF1 (Hayatsu et al., 2000). This suggested that the functional genes for the entire 3M4NP degradation in strain NF100 were separated into at least two non-contiguous gene clusters. However, the genes involved in 3M4NP catabolism in strain SJ98 were clustered together into single gene cluster/operon (*pnpABA1CDEF*). Moreover, the enzymes involved in PNP catabolism are likely not responsible for 3M4NP degradation in strain NF100 (Hayatsu et al., 2000), whereas the catabolism of 3M4NP, PNP, and 2C4NP share the same set of enzymes in strain SJ98 as demonstrated in a previous study and this study. These indicated that strains NF100 and SJ98 likely have different evolutionary patterns in acquiring 3M4NP and PNP catabolic ability.

## Conclusion

*Burkholderia* sp. strain SJ98 degraded 3M4NP via MBQ and MHQ as the intermediates before ring cleavage. Purified PnpA catalyzes the monooxygenation of 3M4NP to MBQ, and *pnpA* is absolutely necessary for the catabolism of 3M4NP. PnpB catalyzes the reduction of MBQ to MHQ. PnpCD, PnpE, and PnpF were involved in the lower pathway of 3M4NP catabolism. These revealed that the enzymes involved in PNP and 2C4NP catabolism were also responsible for 3M4NP degradation in strain SJ98. The present study fills a gap in our understanding of the microbial degradation of 3M4NP at molecular and biochemical levels. Strain SJ98 is capable of degrading PNP and its chloro- and methyl-substituted derivatives, suggesting its potential role in bioremediation of these toxicants-contaminated sites.

## Author Contributions

Conceived and designed the experiments: N-YZ, XH, and JM. Performed the experiments: JM and YL. Analyzed the data: JM, N-YZ, and XH. Wrote the paper: JM, XH, and N-YZ.

## Conflict of Interest Statement

The authors declare that the research was conducted in the absence of any commercial or financial relationships that could be construed as a potential conflict of interest.

## Abbreviations

2C4NP: 2-chloro-4-nitrophenol
3M4NP: 3-methyl-4-nitrophenol
MBQ: methyl-1,4-benzoquinone
MHQ: methylhydroquinone
PNP: *para*-nitrophenol.
